# Lemierre's syndrome with cranial epidural abscess complication: A case report

**DOI:** 10.1016/j.amsu.2022.104478

**Published:** 2022-08-27

**Authors:** Ismail Gedi Ibrahim, Ahmed Adam Osman, Abdinasir Mohamed Elmi, Mahmut Küsbeci, Shuayb Moallim Ali Jama, Abdullahi Yusuf Ali, Faiza Abdulkadir Farah

**Affiliations:** a Department of Radiology Mogadishu Somali Turkish Training and Research Hospital, Mogadishu, Somalia; b Department of Pediatric Surgery Mogadishu Somali Turkish Training and Research Hospital, Mogadishu, Somalia; c Department of ENT Mogadishu Somali Turkish Training and Research Hospital, Mogadishu, Somalia

**Keywords:** Jugular vein thrombosis, Fusobacterium necrophorum, Tonsilitis, Epidural abscess

## Abstract

**Introduction:**

Lemierre's syndrome is a rare disease typically manifested by thrombophlebitis of the jugular vein and septic embolism following a history of oropharyngeal infection. Fusobacterium necrophorum is the causative agent of Lemierre syndrome, commonly known as post-anginal sepsis.

**Case presentation:**

We reported a 24-year-old male who came to the emergency department complaining of a history of a sore throat, fever, malaise, fever, and neck swelling with a normal consciousness level. A laboratory examination showed leukocytosis and high C-reactive protein serum. Radiological diagnosis reveals an anterior neck abscess with left jugular vein thrombosis and left epidural abscess. The blood culture was positive for Fusobacterium necrophorum. The patient underwent surgical drainage and, at the same time, was treated with antibiotics and anticoagulant drugs. After 45 days, the patient improved clinically and was discharged. There were no other symptoms after a one-month follow-up clinically and neck ultrasonography.

**Clinical discussion:**

Lemierre's syndrome has historically had a high mortality rate, approximately up to 90% before antibiotics. The disease's incidence has declined gradually, leading it to become recognized as the “forgotten disease.” Nevertheless, the incidence of Lemierre syndrome has been increasing over the last twenty to thirty years. Primary oropharyngeal infection, bacteremia, radiographic or clinical evidence of internal jugular vein thrombosis, and septic metastatic foci are the main clinical hallmarks of Lemierre's syndrome. Surgical debridement, antibiotics, and anticoagulants are the treatments of choice.

**Conclusion:**

Lemierre's syndrome with cranial epidural abscess is very rare. It is a forgotten disease. Nowadays, the prevalence is increasing. Awareness of clinical and radiological features will aid the prompt management of patients.

## Highlights

• Lemierre's syndrome with cranial epidural abscess is a rare disease that mostly affects young adults and is commonly caused by Fusobacterium necrophorum.
• A clinical manifestation characterized by jugular vein thrombosis and septic embolism following a history of oropharyngeal infection.
• It is a forgotten disease. Nowadays, the prevalence is increasing. Awareness of clinical and radiological features will aid the prompt management of patients.

## 1 Introduction

Lemierre's syndrome is a rare disease typically manifested by thrombophlebitis of the jugular vein and septic embolism following a history of oropharyngeal infection. Fusobacterium necrophorum is the causative agent of Lemierre syndrome, commonly known as post-anginal sepsis [[1], [2]]. Although otogenic, odontogenic, and sinogenic origins have been reported, tonsils and the oropharynx are typically the sites of primary infections [3]. An epidural abscess, spinal abscess, or mediastinitis is an uncommon complication and the most severe [[4], [5]]. We present a young patient with clinical and radiological features of Lemierre syndrome with cranial epidural abscess.

## 2 Case presentation

**Fig. 1:**
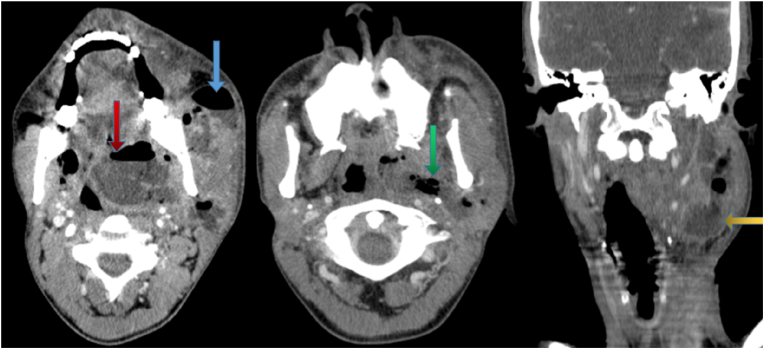
Axial and coronal contrast-enhanced neck Computed tomography reveals soft tissue abscess formation in the peritonsillar, anterior mandibular ramus, and anterior neck region.

**Fig. 2:**
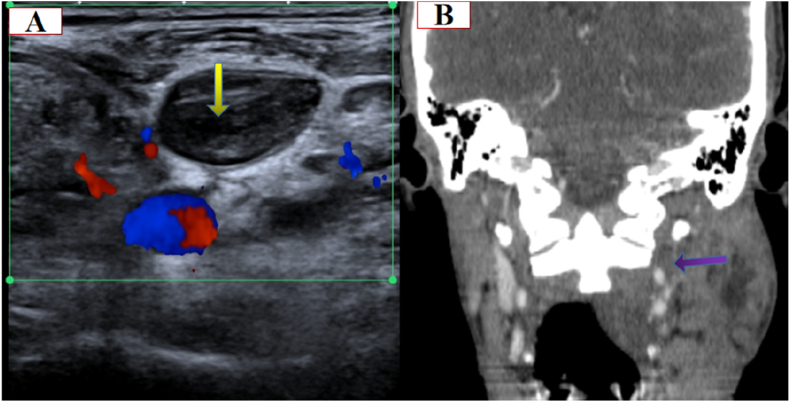
Doppler sonography (A) and contrast-enhanced coronal Computed tomography (B) showed left jugular vein thrombosis.

**Fig. 3:**
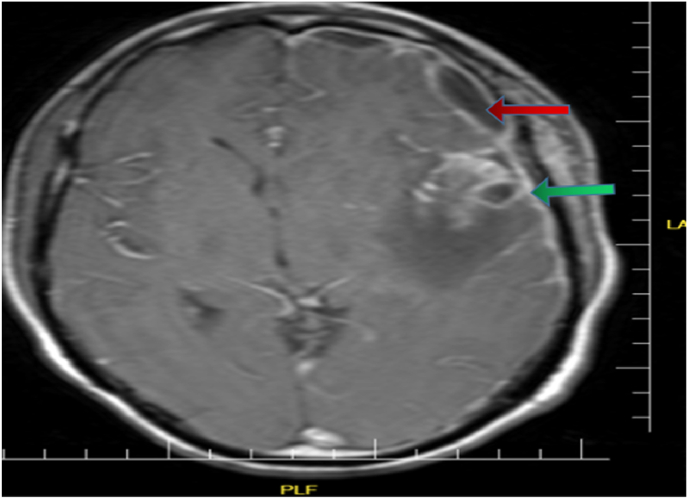
A contrast-enhanced T1 axial MRI showed a cranial epidural abscess. on the left side of the epidural space.

A 24-year-old male comes to the emergency department complaining of a history of sore throat, malaise, fever, and neck and facial swelling with a normal consciousness level. On examination, it reveals tonsilar swelling and tenderness with bilateral cervical lymphadenopathy. The blood pressure and heart rate were normal. The patient had no previous history of chronic disease, chronic drug usage, or allergy. A laboratory examination showed leukocytosis and high C-reactive protein serum. An ultrasound examination demonstrates left jugular vein thrombosis (Fig. 1). Contrast-enhanced neck Computed tomography reveals fluid and air density collections with soft tissue phlegmon formation in the anterior cervical region, in both soft tissue of the anterior mandibular ramus, peritonsillar, left parapharyngeal area, and left carotid space, and also there is left jugular vein thrombosis (Fig. 2). Preliminary differential diagnoses reveal Ludwig's angina and Lemierre's syndrome. The patient underwent surgical drainage in the ENT department and at the same time was treated with Penicillin g 1.2 unit 1 × 1 im for one dose, metrodenzol 3 × 1 iv and unacefen 1g 2 × 1, and Clexane 4000 IU 1 × 1. The blood culture was positive for Fusobacterium necrophorum and was ordered to continue treatment. The patient develops headaches and fever after 10 days. A contrast-enhanced MRI showed an intracranial epidural abscess on the left side of the brain (Fig. 3). The patient was added to the treatment with intravenous clindamycin 600mg 1 × 3 and vancomycin 1g 3 × 1. After 45 days, the patient improved clinically and was discharged for a home treatment regimen with clavulanate 1000 mg 1 × 2; metro 500mg 1 × 3; and warfarin 5 mg 1 × 1. There were no other symptoms after a one-month follow-up clinically and with neck ultrasonography. This work has been reported in line with the SCARE 2020 [12].

## 3 Discussion

Lemierre's syndrome has historically had a high mortality rate, approximately up to 90% before antibiotics. The disease's incidence has declined gradually, leading it to become recognized as the “forgotten disease” [6]. Nevertheless, the incidence of Lemierre syndrome has been increasing over the last twenty to thirty years [7].

Fusobacterium species inhabited in the oral cavity, female vaginal tract, and gastrointestinal tract. The most common species isolated from clinical specimens are F. nucleatum and F. necrophorum. The most common pathogen isolated in patients with Lemierre's syndrome is F necrophorum, which is the more virulent of the two. It's indeed unknown why F necrophorum becomes invasive. One study suggests that nicotine enhances the toxins produced by certain peri-odontopathogens [8].

Primary oropharyngeal infection, bacteremia, radiographic or clinical evidence of internal jugular vein thrombosis, and septic metastatic foci are the main clinical hallmarks of Lemierre's syndrome. A thorough medical history and physical examination are required for diagnosis. In young, otherwise healthy individuals with an underlying oropharyngeal infection who have a worsening clinical course, requiring hospitalization for sepsis and developing pulmonary symptoms in the setting of a recent pharyngeal infection, the syndrome should be suspected. Swelling and discomfort are present in the patient's lateral neck [[9], [10]].

In central nervous system complications, epidural and subdural involvement are the rarest and most serious. Internal jugular vein thrombosis can spread backward into cranial sinuses like the cavernous sinus or sigmoid sinus to the cerebral veins that are upstream from the internal jugular vein sources of septic thrombi. This is a dangerous complication of internal jugular vein thrombosis [[3], [4], [6], [7], [10], [11]].

Contrast-enhanced neck CT examination is the diagnostic tool for Lemierre syndrome. In spite of this, a magnetic resonance imaging scan may be as good as a CT scan for diagnosing Lemierre syndrome [[2], [11], [12]].

Our case comes with a history of sore throat, malaise, fever, and neck and facial swelling. On laboratory examination, the patient had leukocytosis. The radiological examination revealed soft tissue abscesses in the peritonsillar region and anterior neck region, left jugular vein thrombosis, and left brain epidural abscess. The patient was managed with surgical abscess drainage, broad-spectrum antibiotics, and anticoagulants. The case was discharged with clinical improvement and follow-up for home treatment.

## 4 Conclusion

Lemierre's syndrome with cranial epidural abscess is very rare. It is a forgotten disease. Nowadays, the prevalence is increasing. Awareness of clinical and radiological features will aid the prompt management of patients.

## Ethical approval

In our hospital, no ethical approval is required for case reports.

## Sources of funding

There are no sponsors or funding sources for this work.

## Author contribution

Ismail Gedi Ibrahim; written literature review, abstract, and parts of the discussion.

Ahmed Adam Osman: proofreading.

Abdinasir Mohamed Elm: part of the discussion

Mahmut KÜSBECI: Radiological diagnosis.

Shuayb Moalim Ali: introduction.

Abdullahi yusuf ali: Case presentation.

Faiza Abdulkadir Farah: Patients management.

## Registration of research studies

Name of the registry: Not applicable.

Unique identifying number or registration ID: Not applicable.

Hyperlink to your specific registration (must be publicly accessible and will be checked):

## Guarantor

Ismail Gedi Ibrahim MD.

## Declaration of competing interest

Authors have no financial or personal conflict that can influence this work.

## References

[1] Riordan T., Wilson M. (2004). Lemierre's syndrome: more than a historical curiosa. Postgrad. Med..

[2] Kisser U., Gurkov R., Flatz W., Berghaus A., Reichel O. (2012). Lemierre syndrome: a case report. Am J Otolaryngol - Head Neck Med Surg [Internet].

[3] Teng H.W., Chen C.Y., Chen H.C., Chung W.T., Lee W Sen (2012). Fusobacterium septicemia complicated by cerebral subdural and epidural empyemas: a rare case of lemierre syndrome. J Emerg Med [Internet].

[4] Sabaka P., Kachlíková M., Bendžala M., Káčerová H. (2020). Lemierre syndrome caused by Klebsiella pneumoniae complicated by epidural abscess – case report. IDCases.

[5] Peer Mohamed B., Carr L. (2010). Neurological complications in two children with Lemierre syndrome. Dev. Med. Child Neurol..

[6] Meade C.M., Cantos V.D., Nasri H., Serbanescu M., Anderson E.J. (2017). Epidural abscess in Lemierre׳s syndrome. Am J Med Sci [Internet].

[7] Golpe R., Marín B., Alonso M. (1999).

[8] Kushawaha A., Popalzai M., El-Charabaty E., Mobarakai N. (2009). Lemierre's syndrome, reemergence of a forgotten disease: a case report. Cases J.

[9] Riordan T., Wilson M. (2004).

[10] Vargas M.I., Nguyen D., Lovblad K.O. (2010). Radiological approach and findings of septic neurological complications of Lemierre's syndrome. Eur J Radiol Extra [Internet].

[11] Chiu O., Erbay S.H., Bhadelia R.A. (2007). Lemierre's syndrome revisited: case report and imaging findings. Australas. Radiol..

[12] Agha R.A., Franchi T., Sohrabi C., Mathew G., for the SCARE Group (2020). The SCARE 2020 guideline: updating consensus surgical CAse REport (SCARE) guidelines. Int. J. Surg..

